# Association of the Composite dietary antioxidant index with all-cause and cardiovascular mortality: A prospective cohort study

**DOI:** 10.3389/fcvm.2022.993930

**Published:** 2022-10-04

**Authors:** Lei Wang, Zhong Yi

**Affiliations:** ^1^Department of Cardiology, Aerospace Center Hospital, Beijing, China; ^2^Department of Geriatric Medicine, Aerospace Center Hospital, Beijing, China

**Keywords:** composite dietary antioxidant index, all-cause mortality, cardiovascular mortality, healthy diet, oxidative stress

## Abstract

**Background:**

According to epidemiological and experimental data, high individual dietary antioxidant intake is correlated with reduced cancer risk. The correlations between combined dietary antioxidants and the risk of all-cause and cardiovascular mortality remain unclear. Consequently, this study focused on evaluating the correlation between the food-derived Composite Dietary Antioxidant Index (CDAI) and all-cause and cardiovascular mortality.

**Materials and methods:**

Two years of data collected from participants aged ≥20 years were included in this prospective cohort study, which was obtained from the US National Health and Nutrition Examination Survey (NHANES) from 1999 to 2018. The US NHANES adopted a complicated, multistage probability sampling method to collect health data representing the US population. Data collection was done through in-person interviews, virtual physical examinations, and laboratory tests. Mortality-related follow-up statistics from the start of the survey to 31 December 2019 were available. The shape of the correlation between CDAI and all-cause and cardiovascular mortality was inspected using a restricted cubic spline model. For CDAI and all-cause and cardiovascular mortality, the univariate- and multivariate-adjusted Cox proportional hazard models were estimated and presented as regression coefficients and 95% confidence intervals.

**Results:**

In total, 44,031 NHANES participants represented 339.4 million non-institutionalized residents of the US (age, 47.2 ± 16.9 years; 52.5% women, 70.2% non-Hispanic whites, 10.8% non-Hispanic black people, and 7.5% Mexican Americans). In the 118-month follow-up, 9,249 deaths were reported, including 2,406 deaths resulting from heart disease and 519 deaths due to cerebrovascular disease. In the restricted cubic spline regression models, a linear relationship between CDAI and all-cause mortality was present. The weighted multivariate hazard ratios for all-cause mortality were computed to be 0.97 (0.87–1.07) for Q2, 0.88 (0.81–0.96) for Q3, and 0.90 (0.80–1.00) for Q4 (*P* for trend = 0.009) upon comparison with the lowest quartile of CDAI, and an identical trend was observed for cardiovascular mortality.

**Conclusion:**

A high CDAI was linked to decreased all-cause and cardiovascular mortality risk. The intake of an antioxidant-rich diet significantly prevents cardiovascular mortality. To shed more light on these outcomes, more itemized investigations such as randomized control trials are required.

## Background

Cardiovascular disease (CVD) is among the most widely known causes of death (1), accounting for approximately one-third of the deaths around the globe. Moreover, some of the risk factors for CVD include hypertension, dyslipidemia, hyperinsulinemia, hyperglycemia, insulin resistance, and central adiposity (2). Increasing research studies demonstrate that oxidative stress and inflammatory status are closely related to CVD (3–5). Other modifiable risk factors for CVD include diet, physical inactivity, stress, and smoking. Preventing CVD morbidity and mortality has been increasingly prioritized in public health (6) and it is well accepted that a healthy diet helps decrease this burden (7).

Including multiple dietary antioxidants such as manganese, zinc, selenium, and vitamins A and C, the Composite Dietary Antioxidant Index (CDAI) serves as a summary score for reflecting the antioxidant profile of individuals (8, 9). CDAI was constructed in accordance with their aggregate anti-inflammatory impact based on markers like tumor necrosis factor-α and interleukin-1β (10). In addition, because of their critical role in most diets worldwide, interest in the health effects of dietary total antioxidant capacity (TAC) is increasing. Previous studies found an inverse association between CDAI and cancer risk (8, 9, 11); however, similar efforts at cardiovascular mortality among the general population have not yet been conducted.

Epidemiological and experimental studies have indicated the correlation between high individual dietary antioxidant intake and reduced cancer risk. However, the effects of combined dietary antioxidants on the risk of all-cause and cardiovascular mortality remain unclear. Therefore, this research aimed at investigating the link between food-derived CDAI and all-cause and cardiovascular mortality.

## Materials and methods

### Study design and population

This prospective cohort study comprised 2 year data obtained from participants aged ≥20 years at the US National Health and Nutrition Examination Survey (NHANES) from 1999 to 2018. The US NHANES adopted a multistage, complex probability sampling method for collecting health data that represented the US population. The collection of data was done through face-to-face interviews, virtual physical examinations, and lab tests.

### Composite dietary antioxidant index calculation

All NHANES participant are eligible for two 24-hourh dietary recall interviews. Interview data files were sent electronically from the field and were imported into Survey Net, a computer-assisted food coding and data management system developed. Two types of dietary intake data are available on the NHANES website: Individual Foods files and Total Nutrient Intakes files. The study draws upon data from the Total Nutrient Intakes Files: For each participant, daily total energy and nutrient intakes from foods and beverages. In the case of vitamin A, the NHANES website provides the intake of vitamin A (mg). CDAI development has been described (9) and validated in a previous report (10). To estimate CDAI, we standardized each of the same six dietary vitamins and minerals (vitamins A, C, and E, selenium, manganese, and zinc from food only) by subtracting the global mean and dividing by the global standard deviation. We then calculated the CDAI by summing the standardized intakes of these vitamins and minerals with equal weight, as described next:


$$\mathrm{CDAI}=\sum_{i=1}^{6}\frac{\mathrm{Xi}-\mu \,i}{\mathrm{Si}}$$

*Xi* represented the everyday antioxidant intake *i*; μ*i* represented the mean *Xi* of the whole cohort for antioxidants *i*; *Si* represented the standard deviation (SD) for μ *i*.

### Baseline data collection

The information on covariates was obtained by means of baseline questionnaires. These questionnaires comprised questions on age, gender, level of literacy, marital status, smoking status, race/ethnicity, the income-poverty ratio of their family, and self-reported baseline medical history, such as diabetes, myocardial infarction, hypertension, hypercholesterolemia, CVD, stroke, and chronic bronchitis, and medications such as antihypertensives, hypoglycaemic agents, and lipid-lowering medications. The body mass index was derived by the measurements of height and weight and other laboratory measurements were performed in accordance with the laboratory procedure manual for NHANES. The NHANES instructions for operations^1^ describe the methodologies and processes employed for study visits and collecting clinical laboratory information. The diagnostic criteria for smoking were as follows: never smoked, smoked less than 100 cigarettes throughout life; former smoker, smoked more than 100 cigarettes throughout life and has currently given up smoking, the active smoker at present, smoked more than 100 cigarettes throughout life and smokes on some days or every day at present. On the other hand, the diagnostic criteria for the consumption of alcohol were as follows: heavy drinking at present (≥4 drinks every day for males, ≥3 drinks every day for females, or ≥5 days per month of binge drinking), moderate alcohol user at present (≥3 drinks every day for males, ≥2 drinks every day for females, or ≥2 days per month of binge drinking), or current mild alcohol user (one who does not meet the above criteria).

Diabetes was categorized as follows: diabetes mellitus (DM), compromised fasting glycemia, or impaired glucose tolerance, and the following were the diagnostic criteria for it (12): self-reported diagnosis of diabetes, use of medication to treat diabetes or insulin, hemoglobin A1c level of ≥6.5%, fasting plasma glucose of ≥7.0 mmol/l (126 mg/dl), random plasma blood glucose of ≥11.1 mmol/l (200 mg/dl), 2 h oral glucose tolerance test blood sugar of ≥11.1 mmol/l (200 mg/dl). The following stated were the diagnostic criteria for hypertension (13, 14): self-reported diagnosis of hypertension, intake of antihypertensive medications, or average systolic blood pressure of ≥140 mmHg and/or average diastolic blood pressure of ≥90 mmHg. The average blood pressure was calculated as follows: Diastolic average was not calculated when the diastolic reading was zero. The average would be zero when all diastolic readings were zero. In the case of obtaining only one blood pressure reading, it was considered the average. In the case of more than one blood pressure reading, we always eliminated the first reading from the average.

The CVD status of participants was measured by a self-reported diagnosis of at least one of the five subtypes of CVD: coronary artery disease, angina, myocardial infarction, congestive heart failure, and stroke. It was detected by self-reported positive selection (yes/no) for at least one of these health conditions, and people with CVD may fall under more than one CVD subtype.

Chronic kidney disease was defined as aberrant kidney function based on the Kidney Disease: Improving Global Outcomes 2021 clinical practice guidelines (15).

### Mortality

The NHANES-assigned sequence number was used to link de-identified and anonymized participant data from 1999 to 2018 to longitudinal Medicare and mortality data. There were mortality follow-up statistics from the beginning of the survey until 31 December 2019 were available. We evaluated all-cause mortality and mortality linked to cardiac diseases (I00–I09, I11, I13, and I20–I51), Alzheimer’s (G30), DM (E10–E14), chronic lower respiratory diseases (J40–J47), malignant neoplasms (C00–C97), cerebrovascular diseases (I60–I69), and other causes. Cardiovascular mortality was demonstrated by means of the 10th revision of the International Classification of Diseases (ICD-10). It also included deaths from heart diseases (I00-I09, I11, I13, and I20-I51) and cerebrovascular diseases (I60–169) and it also helped in identifying the death cause.

### Statistical analysis

Continuous variables are presented as the mean ± SD (Gaussian distribution) or median (range; Skewed distribution), and categorical variables are reported as numbers (%). χ^2^ (categorical variable), the one-way analysis of variance test (normal distribution), or the Kruskal–Wallis H test (skewed distribution) were utilized to test the variations among the CDAI quartiles. To account for multiple testing, two-sided *p*-values were adjusted according to the method of Benjamini/Hochberg (B/H) to control the false discovery rate (FDR). An association was considered to be statistically significant, if its corresponding B/H-adjusted *p*-value was below 0.05, corresponding to an FDR of 5%. Before data analysis, the variables were inspected for missing values. The proportion of missing data was 0.00–37.5% (alcohol use) and to indicate missing covariate values to include these data from the analyses, dummy variables were adopted. The shape of the correlation between CDAI and all-cause and cardiovascular mortality was examined using a restricted cubic spline model. We selected three knots at the 25th, 50th, and 75th quartiles. Further, univariate- and multivariate-adjusted Cox proportional hazard models were estimated for CDAI, and the findings are presented as regression coefficients and 95% confidence intervals (CIs). The regression models were estimated for the entire sample and adjusted for demographics, socioeconomics, behavior, anthropometric variables, and medical history. *P* < 0.05 was considered a criterion of a significant difference.

### Sensitivity analysis

We performed subgroup analyses by gender (male or female), age (<65 or ≥65 years) at cohort entry, and medical history (diabetes and hypertension). Multivariate-adjusted Cox proportional hazard models were estimated for CDAI, and the findings were presented as regression coefficients and 95% CIs. Additionally, the statistical package for all statistical analyses, R 4.1.2^2^ was employed and it was modified for complex survey design and population weighting following survey protocols. The outcomes could be applied and extrapolated to the entire adult population of the US by incorporating population weights, stratum variables, and main sampling units into the analysis, accounting for differential probability of inclusion into the sample and non-response bias.

## Results

In total, 44,031 NHANES participants represented 339.4 million non-institutionalized residents of the US (age, 47.2 ± 16.9 years; 52.5% women, 70.2% non-Hispanic whites, 10.8% non-Hispanic black people, and 7.5% Mexican Americans) (Table 1).

**TABLE 1:** Weighted characteristics of the study participants by the quartiles of composite dietary antioxidant index.

	Level	Overall	Q1	Q2	Q3	Q4	Unadjusted *p*-value	Adjusted *p*-value
								
CDAI			<−2.1	−2.1∼0.1	0.1∼2.7	≥2.7		
								
N		339355711	110316384	78007777	75808160	75223390		
Age [mean (SD)]		47.2 (16.9)	47.1 (17.4)	47.6 (17.0)	47.6 (16.8)	46.4 (16.1)	<0.001	<0.001
Sex (%)	Female	178121841 (52.5)	69701502 (63.2)	44692798 (57.3)	38963486 (51.4)	24764055 (32.9)	<0.001	<0.001
	Male	161233870 (47.5)	40614882 (36.8)	33314978 (42.7)	36844674 (48.6)	50459336 (67.1)		
BMI [mean (SD)]		28.7 (6.7)	28.5 (6.6)	28.8 (6.8)	28.8 (6.6)	28.7 (6.6)	0.03	0.03
Race/ethnicity (%)	Mexican American	25572639 (7.5)	7636053 (6.9)	5835120 (7.5)	5746815 (7.6)	6354651 (8.4)	0.013	0.014
	Non-Hispanic Black	36605626 (10.8)	13804486 (12.5)	8889082 (11.4)	7433325 (9.8)	6478733 (8.6)		
	Non-Hispanic White	238058699 (70.2)	75506501 (68.4)	54624888 (70.0)	54166517 (71.5)	53760793 (71.5)		
	Other Race	39118747 (11.5)	13369344 (12.1)	8658687 (11.1)	8461503 (11.2)	8629213 (11.5)		
Education (%)	College or above	196527930 (57.9)	52984625 (48.0)	44474453 (57.0)	48619421 (64.1)	50449431 (67.1)	<0.001	<0.001
	High school or equivalent	122634881 (36.1)	48122613 (43.6)	28976276 (37.1)	23603931 (31.1)	21932062 (29.2)		
	Less than high school	19835061 (5.8)	9041536 (8.2)	4467900 (5.7)	3498074 (4.6)	2827552 (3.8)		
Marital status (%)	Married	191403497 (56.4)	58327826 (52.9)	43173816 (55.3)	44934453 (59.3)	44967403 (59.8)	<0.001	<0.001
	Never married	79194193 (23.3)	24987044 (22.7)	17785773 (22.8)	17411274 (23.0)	19010102 (25.3)		
	Separated	61357353 (18.1)	22794026 (20.7)	15047938 (19.3)	12621649 (16.6)	10893740 (14.5)		
Family income-poverty ratio [mean (SD)]		3.02 (1.63)	2.73 (1.63)	2.98 (1.61)	3.18 (1.60)	3.30 (1.61)	<0.001	<0.001
Family income-poverty ratio (%)	<1.0	42588716 (12.5)	17400946 (15.8)	9799126 (12.6)	7949230 (10.5)	7439413 (9.9)	<0.001	<0.001
	1.0–3.0	114663927 (33.8)	40518143 (36.7)	26940266 (34.5)	24501539 (32.3)	22703979 (30.2)		
	>3.0	157896137 (46.5)	43045746 (39.0)	35810803 (45.9)	38420252 (50.7)	40619337 (54.0)		
Smoking status (%)	Never	180442803 (53.2)	54681255 (49.6)	41735486 (53.5)	42416503 (56.0)	41609559 (55.3)	<0.001	<0.001
	Former	86304571 (25.4)	25708560 (23.3)	19371132 (24.8)	19791775 (26.1)	21433104 (28.5)		
	Now	72359853 (21.3)	29851348 (27.1)	16812181 (21.6)	13545232 (17.9)	12151094 (16.2)		
Alcohol use (%)	Mild	115551843 (34.1)	31978793 (29.0)	26812016 (34.4)	27688346 (36.5)	29072688 (38.6)	<0.001	<0.001
	Moderate	59170559 (17.4)	18474581 (16.7)	13181019 (16.9)	14105120 (18.6)	13409840 (17.8)		
	Heavy	57988126 (17.1)	19286772 (17.5)	13736056 (17.6)	12114479 (16.0)	12850819 (17.1)		
DM (%)	No	273492729 (80.6)	91809507 (83.2)	62255113 (79.8)	59563164 (78.6)	59864945 (79.6)	<0.001	<0.001
	DM	40600421 (12.0)	12316058 (11.2)	10045433 (12.9)	9509844 (12.5)	8729085 (11.6)		
	IFG	13285821 (3.9)	3805847 (3.4)	3077285 (3.9)	3131930 (4.1)	3270760 (4.3)		
	IGT	6531440 (1.9)	635958 (0.6)	1560781 (2.0)	2233202 (2.9)	2101499 (2.8)		
Hypertension (%)	No	214176789 (63.1)	69673740 (63.2)	47652510 (61.1)	48074949 (63.4)	48775590 (64.8)	0.003	0.003
	Yes	125178922 (36.9)	40642644 (36.8)	30355267 (38.9)	27733211 (36.6)	26447800 (35.2)		
CVD (%)	No	309384087 (91.2)	99233824 (90.0)	70816311 (90.8)	69306045 (91.4)	70027907 (93.1)	<0.001	<0.001
	Yes	29949235 (8.8)	11079480 (10.0)	7187320 (9.2)	6501139 (8.6)	5181296 (6.9)		
Hyperlipidemia (%)	No	101630847 (29.9)	31450627 (28.5)	22522172 (28.9)	22998184 (30.3)	24659863 (32.8)	<0.001	<0.001
	Yes	237677859 (70.0)	78860965 (71.5)	55451892 (71.1)	52803332 (69.7)	50561671 (67.2)		
CKD (%)	No	152237701 (44.9)	16582761 (15.0)	35132488 (45.0)	47522497 (62.7)	52999956 (70.5)	<0.001	<0.001
	Yes	26766362 (7.9)	3981582 (3.6)	7774760 (10.0)	7743420 (10.2)	7266601 (9.7)		

Data are presented as frequencies (percentages) or mean (SD) or median (IQR). Numbers that do not add up to 100% are attributable to missing data. CDAI, composite dietary antioxidant index; BMI, the body-mass index is determined as follows: the weight in kilograms (Kgs)/(height in square meters (m^2^); DM, diabetes mellitus; IFG, impaired fasting glycaemia; IGT, impaired glucose tolerance; CVD, cardiovascular disease; CKD, chronic kidney disease.

Table 1 provides weighted baseline features of those who participated in this study stratified by the CDAI quartiles. A significant age difference was observed between the CDAI quartiles (*P* < 0.001) (Supplementary Table 1); the participants in the fourth quartile were younger (46.4 ± 16.1 years). Moreover, male predilection was observed in the fourth quartile, whereas female predilection was observed in the first quartile (*P* < 0.001). The ratio of non-Hispanic whites was high in the third and fourth quartiles (71.5%, *P* = 0.013). In the first quartile, there were more married participants (59.8%, *P* < 0.001) and individuals with higher family income-poverty ratios belonged to the fourth quartile (*P* < 0.001). Most participants with a “college or above” level of education belonged to the fourth quartile (67.1%, *P* < 0.001), and the proportion of pre-existing CVD at baseline was higher among the participants in the first quartile (10.0%, *P* < 0.001).

**FIGURE 1:**
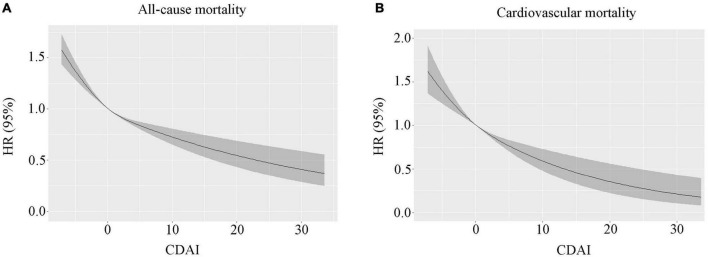
**(A)** The correlation between the continuous CDAI and all-cause mortality was demonstrated using generalized additive models. **(B)** The correlation between continuous CDAI and cardiovascular mortality is demonstrated using generalized additive models. CDAI, composite dietary antioxidant index.

In the 118-month follow-up, 9,249 deaths were reported, including 2,406 deaths resulting from cardiac disease and 519 deaths resulting from cerebrovascular disease. In the restricted cubic spline regression models, the relationship between CDAI and all-cause mortality was linear (Figure 1).

Within the weighted unadjusted model, the link between CDAI and all-cause mortality was 0.95 (0.94–0.96) (Supplementary Table 2). Higher CDAI was linked to a reduced risk of all-cause mortality after controlling for demographics, socioeconomics, behavior, anthropometric variables, medical history, medications, and laboratory findings (Model 5). When comparing with the lowest CDAI quartile, the weighted multivariate hazard ratios (HRs) for all-cause mortality were 0.97 (0.87–1.07) for Q2, 0.88 (0.81–0.96) for Q3, and 0.90 (0.80–1.00) for Q4 (*P* for trend = 0.009) (Table 2).

**TABLE 2:** Weighted associations between the CDAI and all-cause and cardiovascular mortality in the multivariable, and crude analyses.

	Q1	Q2	Q3	Q4	*P*-value for trend
CDAI	<−2.1	−2.1∼0.1	0.1∼2.7	≥2.7	
**All-cause mortality**					
Model 1	1	0.90 (0.82–0.99) 0.024	0.74 (0.69–0.81) < 0.001	0.65 (0.59–0.72) < 0.001	<0.001
Model 2	1	0.88 (0.80–0.97) 0.008	0.72 (0.66–0.78) < 0.001	0.61 (0.55–0.68) < 0.001	<0.001
Model 3	1	0.87 (0.80–0.95) 0.001	0.73 (0.67–0.79) < 0.001	0.69 (0.63–0.76) < 0.001	<0.001
Model 4	1	0.94 (0.86–1.02) 0.135	0.80 (0.74–0.87) < 0.001	0.78 (0.70–0.85) < 0.001	<0.001
Model 5	1	0.97 (0.87–1.07) 0.485	0.88 (0.81–0.96) 0.003	0.90 (0.80–1.00) 0.057	0.009
**Cardiovascular mortality**					
Model 1	1	0.87 (0.77–0.98) 0.020	0.73 (0.62–0.86) < 0.001	0.58 (0.48–0.69) < 0.001	<0.001
Model 2	1	0.84 (0.74–0.95) 0.006	0.70 (0.60–0.83) < 0.001	0.53 (0.44–0.64) < 0.001	<0.001
Model 3	1	0.84 (0.73–0.97) 0.014	0.72 (0.61–0.86) < 0.001	0.62 (0.51–0.75) < 0.001	<0.001
Model 4	1	0.90 (0.79–1.04) 0.154	0.79 (0.67–0.94) 0.008	0.70 (0.58–0.84) < 0.001	<0.001
Model 5	1	0.94 (0.81–1.08) 0.368	0.86 (0.72–1.03) 0.108	0.81 (0.66–0.99) 0.040	0.024

Data are hazard ratio (95% CI). Model 1 unadjusted. Model 2 adjusted for sex. Model 3 adjusted for age, sex. Model 4 adjusted for model 3 covariates plus ethnicity, family income-poverty ratio level, education, and marital status. Model 5 adjusted for model 4 covariates plus smoking status, diabetes, hypertension, CVD, and CKD. HR, hazard ratio; CI, confidence interval; CDAI, composite dietary antioxidant index; CVD, cardiovascular disease; CKD, chronic kidney disease.

In the restricted cubic spline regression models, the relationship between CDAI and cardiovascular mortality was linear (Figure 1B). Within the weighted unadjusted model, the link between CDAI and CVD mortality was 0.94 (0.92–0.96) (Supplementary Table 3). Higher CDAI was linked to a lower risk of cardiovascular mortality after controlling for demographics, socioeconomics, behavior, anthropometric variables, medical history, medications, and laboratory findings (Model 5). When comparing with the lowest CDAI quartile, the weighted multivariate HRs for cardiovascular mortality were 0.94 (0.81–1.08) for Q2, 0.86 (0.72–1.03) for Q3, and 0.81 (0.66–0.99) for Q4 (*P* for trend = 0.024) (Table 2).

### Sensitivity analysis

We performed subgroup analyses to stratify the link between CDAI and all-cause and cardiovascular mortality by age, gender, and medical history, as demonstrated in Supplementary Table 4. No interaction was observed between the subgroup variables and the link between CDAI with all-cause and cardiovascular mortality.

## Discussion

In this population-based cohort study, we observed that CDAI was negatively linked to all-cause mortality, and people in the highest CDAI quartile had a reduced risk of all-cause mortality in comparison to those in the lowest quartile, consistent with the trends of cardiovascular mortality. Although diet quality index scores (DQIS) are used for predicting health outcomes traditionally as mentioned in this abstract, dietary TAC also can be used as a novel tool to predict the total antioxidant potential present in the diet. Furthermore, Kyungho proposes that dietary TAC might possess a valid ability to predict all-cause mortality in the US population rather than the DQIS (16). As the determination of TAC is based on the capacity of the plasma assay to reduce ferric, it does have the limitation of only focussing on one aspect of the antioxidant activity *in vivo*. The absence of a “gold standard” for assessing TAC determined the assay limitation of the database with antioxidant capacity values. Moreover, the above database cannot be compared with another that uses a different TAC assay. The CDAI is a summary score of multiple dietary antioxidants representing an individual’s antioxidant profile, resulting in increased applicability.

During aging, different levels of cell injury can be caused as a result of oxidative reactions, thus mediating the pathogenesis of chronic diseases, such as CVD, atherosclerosis, and cancer. As an external contributor, diet can regulate the plasma redox state and protect against reactive oxygen and nitrogen species. Dietary TAC has an impact on the population with a cardiometabolic risk profile (17–19). An evaluation of the link between dietary TAC and CVD risk factors based on a systematic review of observational studies revealed inverse relationships for fasting blood sugar, blood pressure, C-reactive protein, and waist circumference and positive relationships for high-density lipoprotein cholesterol (20). A study demonstrated that high dietary TAC was significantly associated with a reduced odds ratio for the prevalence of metabolic syndrome components, such as elevated blood pressure and diabetes (21, 22). Additionally, a high dietary TAC was related to a decreased risk of non-alcoholic fatty liver disease (23, 24). According to Mahdieh et al., the dietary antioxidant quality negatively predicted death among individuals who received coronary artery bypass grafting (25). The estimation of the CDAI provides further information because of its ability to identify and classify the possible antioxidant sources in complex diets, thereby sorting out diets and individuals in accordance with their intake of antioxidants. This makes CDAI beneficial in nutritional epidemiology studies and CDAI can be used to plan actions to promote better nutrition, which can be achieved by implementing dietary antioxidant interventions to contribute to improving eating habits and lifestyles.

### Limitations

The present research has certain limitations. It is an observational study that did not have data that highlighted the use of any dietary supplements and behavioral (physical activity and sleep) changes. The diagnosis of CVD based on the self-reports from participants, this may lead to discrepancies from the actual situation. Moreover, the CDAI was derived only at baseline and the accuracy to reflect the long-term consumption patterns may be compromised.

## Conclusion

A high CDAI was correlated with a decreased risk of all-cause and cardiovascular mortality. The intake of a diet high in antioxidants significantly prevents cardiovascular mortality. Increasingly itemized investigations such as randomized control trials are warranted to reveal more insight into these results.

## Data availability statement

The raw data supporting the conclusions of this article will be made available by the authors, without undue reservation.

## Ethics statement

Ethical review and approval was not required for the study on human participants in accordance with the local legislation and institutional requirements. The patients/participants provided their written informed consent to participate in this study.

## Author contributions

Both authors made contributions to data collection, analysis and interpretation of the data, and drafted and reviewed the manuscript.

## Footnotes

1
https://wwwn.cdc.gov/nchs/nhanes/analyticguidelines.aspx

2
http://www.r-project.org
