# Supplementary material for: Association of the Composite dietary antioxidant index with all-cause and cardiovascular mortality: A prospective cohort study
Source: Front Cardiovasc Med. 2022 Oct 4;9:993930. doi: 10.3389/fcvm.2022.993930 (PMC9577254; doi:10.3389/fcvm.2022.993930)
Supplement: Supplementary file 1 [file Data_Sheet_1.doc]

Supplemental Material

| CDAI |  | Q1 | Q2 | Q3 | Q4 |
| --- | --- | --- | --- | --- | --- |
| Age | Q1 |  | 1 | <0.001 | <0.001 |
|  | Q2 |  |  | <0.001 | <0.001 |
|  | Q3 |  |  |  | <0.001 |
| Sex (%) | Q1 |  | 1 | <0.001 | <0.001 |
|  | Q2 |  |  | <0.001 | <0.001 |
|  | Q3 |  |  |  | <0.001 |
| BMI | Q1 |  | 0.002 | 1 | 0.005 |
|  | Q2 |  |  | 0.097 | <0.001 |
|  | Q3 |  |  |  | <0.001 |
| Race/ethnicity (%) | Q1 |  | <0.001 | <0.001 | <0.001 |
|  | Q2 |  |  | <0.001 | <0.001 |
|  | Q3 |  |  |  | <0.001 |
| Education (%) | Q1 |  | <0.001 | <0.001 | <0.001 |
|  | Q2 |  |  | <0.001 | <0.001 |
|  | Q3 |  |  |  | <0.001 |
| Marital status (%) | Q1 |  | <0.001 | <0.001 | <0.001 |
|  | Q2 |  |  | <0.001 | <0.001 |
|  | Q3 |  |  |  | <0.001 |
| Family income-poverty ratio | Q1 |  | <0.001 | <0.001 | <0.001 |
|  | Q2 |  |  | <0.001 | <0.001 |
|  | Q3 |  |  |  | <0.001 |
| Family income-poverty ratio (%) | Q1 |  | <0.001 | <0.001 | <0.001 |
|  | Q2 |  |  | <0.001 | <0.001 |
|  | Q3 |  |  |  | <0.001 |
| Smoking status (%) | Q1 |  | <0.001 | <0.001 | <0.001 |
|  | Q2 |  |  | <0.001 | <0.001 |
|  | Q3 |  |  |  | <0.001 |
| Alcohol use (%) | Q1 |  | <0.001 | <0.001 | <0.001 |
|  | Q2 |  |  | <0.001 | <0.001 |
|  | Q3 |  |  |  | <0.001 |
| DM (%) | Q1 |  | <0.001 | <0.001 | <0.001 |
|  | Q2 |  |  | <0.001 | <0.001 |
|  | Q3 |  |  |  | <0.001 |
| Hypertension (%) | Q1 |  | <0.001 | <0.001 | <0.001 |
|  | Q2 |  |  | <0.001 | <0.001 |
|  | Q3 |  |  |  | <0.001 |
| CVD (%) | Q1 |  | <0.001 | <0.001 | <0.001 |
|  | Q2 |  |  | <0.001 | <0.001 |
|  | Q3 |  |  |  | <0.001 |
| Hyperlipidemia (%) | Q1 |  | <0.001 | <0.001 | <0.001 |
|  | Q2 |  |  | <0.001 | <0.001 |
|  | Q3 |  |  |  | <0.001 |
| CKD (%) | Q1 |  | <0.001 | <0.001 | <0.001 |
|  | Q2 |  |  | <0.001 | <0.001 |
|  | Q3 |  |  |  | <0.001 |

Table S1. Weighted pair-wise comparison among the CDAI quartiles

Data are presented as adjusted p value according to Bonferroni.

Abbreviations:

CDAI, composite dietary antioxidant index

BMI, the body-mass index is determined as follows: the weight in kilograms (Kgs) / (height in square meters (m^2^)

DM, diabetes mellitus

IFG, impaired fasting glycaemia

IGT impaired glucose tolerance

CVD, cardiovascular disease

CKD, chronic kidney disease

|  | Level | HR (95%CI) | P |
| --- | --- | --- | --- |
| Age |  | 1.09 (1.09,1.10) | <0.001 |
| Sex | Female | 1 |  |
|  | Male | 1.15 (1.08,1.21) | <0.001 |
| Race/ethnicity | Mexican American | 1 |  |
|  | Non-Hispanic Black | 2.23 (1.92,2.60) | <0.001 |
|  | Non-Hispanic White | 2.44 (2.12,2.81) | <0.001 |
|  | Other Race | 1.42 (1.15,1.75) | <0.001 |
| Education | College or above | 1 |  |
|  | High school or equivalent | 1.80 (1.68,1.92) | <0.001 |
|  | Less than high school | 3.17 (2.85,3.53) | <0.001 |
| Marital status | Married | 1 |  |
|  | Never married | 0.51 (0.45,0.58) | <0.001 |
|  | Separated | 2.37 (2.18,2.58) | <0.001 |
| Family income-poverty ratio | <1.0 | 1 |  |
|  | 1.0-3.0 | 1.15 (1.05,1.26) | <0.001 |
|  | >3.0 | 0.56 (0.50,0.63) | <0.001 |
| CDAI |  | 0.95 (0.94,0.96) | <0.001 |
| BMI |  | 1.18 (1.12,1.25) | <0.001 |
| Smoking status | Never | 1 |  |
|  | Former | 2.08 (1.91,2.21) | <0.001 |
|  | Now | 1.42 (1.28,1.56) | <0.001 |
| Alcohol use | Mild | 1 |  |
|  | Moderate | 0.61 (0.54,0.69) | <0.001 |
|  | Heavy | 0.58 (0.50,0.67) | <0.001 |
| DM | No | 1 |  |
|  | DM | 3.79 (3.50,4.11) | <0.001 |
|  | IFG | 2.36 (2.09,2.61) | <0.001 |
|  | IGT | 1.81 (1.53,2.15) | <0.001 |
| Hypertension |  | 4.26 (4.01,4.54) | <0.001 |
| Hyperlipidemia |  | 1.63 (1.51,1.75) | <0.001 |
| CVD |  | 6.04 (5.54, 6.58) | <0.001 |
| CKD |  | 6.42 (5.94,6.95) | <0.001 |

Table S2. Weighted univariate cox regression model for all-cause mortality

Abbreviations:

HR, hazard ratio

CI, confidence interval

CDAI, composite dietary antioxidant index

BMI, the body-mass index is determined as follows: the weight in kilograms (Kgs) / (height in square meters (m^2^)

DM, diabetes mellitus

IFG, impaired fasting glycaemia

IGT impaired glucose tolerance

CVD, cardiovascular disease

CKD, chronic kidney disease

|  | Level | HR (95%CI) | P |
| --- | --- | --- | --- |
| Age |  | 1.11 (1.11,1.12) | <0.001 |
| Sex | Female | 1 |  |
|  | Male | 1.21 (1.09,1.35) | <0.001 |
| Race/ethnicity | Mexican American | 1 |  |
|  | Non-Hispanic Black | 2.61 (2.07,3.29) | <0.001 |
|  | Non-Hispanic White | 2.73 (2.20,3.39) | <0.001 |
|  | Other Race | 1.37 (1.01,1.84) | 0.040 |
| Education | College or above | 1 |  |
|  | High school or equivalent | 1.84 (1.61,2.11) | <0.001 |
|  | Less than high school | 3.71 (3.08,4.46) | <0.001 |
| Marital status | Married | 1 |  |
|  | Never married | 0.44 (0.36,0.54) | <0.001 |
|  | Separated | 2.64 (2.27,3.06) | <0.001 |
| Family income-poverty ratio | <1.0 | 1 |  |
|  | 1.0-3.0 | 1.35 (1.11,1.64) | <0.001 |
|  | >3.0 | 0.56 (0.46,0.67) | <0.001 |
| CDAI |  | 0.94 (0.92,0.96) | <0.001 |
| BMI |  | 1.33 (1.22,1.46) | <0.001 |
| Smoking status | Never | 1 |  |
|  | Former | 1.91 (1.68,2.17) | <0.001 |
|  | Now | 1.00 (0.84,1.19) | 0.985 |
| Alcohol use | Mild | 1 |  |
|  | Moderate | 0.48 (0.39,0.60) | <0.001 |
|  | Heavy | 0.34 (0.26,0.44) | <0.001 |
| DM | No | 1 |  |
|  | DM | 4.72 (4.16,5.37) | <0.001 |
|  | IFG | 2.70 (2.10,3.48) | <0.001 |
|  | IGT | 1.87 (1.38,2.52) | <0.001 |
| Hypertension |  | 6.33 (5.46,7.33) | <0.001 |
| Hyperlipidemia |  | 1.91 (1.63,2.24) | <0.001 |
| CVD |  | 9.80 (8.55,11.24) | <0.001 |
| CKD |  | 8.96 (7.41,10.83) | <0.001 |

Table S3. Weighted univariate cox regression model for cardiovascular mortality

Abbreviations:

HR, hazard ratio

CI, confidence interval

CDAI, composite dietary antioxidant index

BMI, the body-mass index is determined as follows: the weight in kilograms (Kgs) / (height in square meters (m^2^)

DM, diabetes mellitus

IFG, impaired fasting glycaemia

IGT impaired glucose tolerance

CVD, cardiovascular disease

CKD, chronic kidney disease

|  | Q1 | Q2 | Q3 | Q4 | *P* Value for Trend | *P* Value for Interaction |
| --- | --- | --- | --- | --- | --- | --- |
| CDAI | < -2.1 | -2.1~0.1 | 0.1~2.7 | ≥2.7 |  |  |
| All- cause mortality |  |  |  |  |  |  |
| Age, years |  |  |  |  |  |  |
| ≥65 | 1 | 0.92 (0.82-1.04) 0.197 | 0.89 (0.78-1.01) 0.075 | 0.88 (0.77-1.00) 0.057 | 0.038 | 0.076 |
| <65 | 1 | 0.93 (0.77-1.12) 0.436 | 0.77 (0.65-0.91) 0.003 | 0.78(0.65-0.94) 0.009 | 0.001 |  |
| Sex |  |  |  |  |  |  |
| Female | 1 | 0.97 (0.86-1.10) 0.664 | 0.87 (0.77-0.99) 0.036 | 0.87 (0.73-1.04) 0.136 | 0.050 | 0.446 |
| Male | 1 | 0.96 (0.84-1.10) 0.589 | 0.87 (0.77-0.97) 0.016 | 0.89 (0.78-1.02) 0.097 | 0.031 |  |
| Diabetes |  |  |  |  |  |  |
| No | 1 | 0.92 (0.82-1.04) 0.168 | 0.82 (0.73-0.92) 0.001 | 0.84 (0.72-0.97) 0.017 | 0.002 | 0.430 |
| Yes | 1 | 1.01 (0.84-1.21) 0.952 | 0.93 (0.77-1.11) 0.403 | 0.92 (0.74-1.13) 0.413 | 0.383 |  |
| Hypertension |  |  |  |  |  |  |
| No | 1 | 0.92 (0.75-1.13) 0.437 | 0.86 (0.73-1.02) 0.078 | 0.88 (0.73-1.07) 0.199 | 0.092 | 0.316 |
| Yes | 1 | 0.98 (0.89-1.08) 0.645 | 0.86 (0.77-0.96) 0.006 | 0.86 (0.76-0.99) 0.029 | 0.009 |  |
| Cardiovascular mortality |  |  |  |  |  |  |
| Age, years |  |  |  |  |  |  |
| ≥65 | 1 | 0.87 (0.73-1.04) 0.380 | 0.85 (0.68-1.05) 0.127 | 0.79 (0.62-1.00) 0.050 | 0.039 | 0.053 |
| <65 | 1 | 0.95 (0.72-1.25) 0.436 | 0.80 (0.56-1.14) 0.219 | 0.73 (0.49-1.08) 0.117 | 0.085 |  |
| Sex |  |  |  |  |  |  |
| Female | 1 | 1.01 (0.85-1.20) 0.912 | 0.95 (0.76-1.17) 0.610 | 0.80 (0.58-1.10) 0.175 | 0.229 | 0.855 |
| Male | 1 | 0.87 (0.71-1.06) 0.168 | 0.78 (0.61-1.00) 0.048 | 0.77 (0.59-1.01) 0.061 | 0.032 |  |
| Diabetes |  |  |  |  |  |  |
| No | 1 | 0.94 (0.76-1.17) 0.582 | 0.86 (0.67-1.10) 0.238 | 0.80 (0.61-1.04) 0.100 | 0.095 | 0.134 |
| Yes | 1 | 0.83 (0.65-1.07) 0.148 | 0.80 (0.61-1.05) 0.109 | 0.70 (0.50-0.98) 0.035 | 0.028 |  |
| Hypertension |  |  |  |  |  |  |
| No | 1 | 0.92 (0.64-1.31) 0.656 | 0.88 (0.61-1.25) 0.467 | 0.80 (0.51-1.24) 0.315 | 0.227 | 0.871 |
| Yes | 1 | 0.94 (0.80-1.10) 0.442 | 0.85 (0.69-1.05) 0.130 | 0.80 (0.65-1.00) 0.047 | 0.015 |  |

Table S4. Weighted multivariable-adjusted hazard ratios for the association between quartiles of CDAI and all-cause mortality and cardiovascular mortality by subgroups.

Data are hazard ratio (95% CI)

Models are adjusted for age, sex, ethnicity, family income-poverty ratio level, education, marital status, smoking status, diabetes, hypertension, CVD, and CKD except the subgroup variable itself.

Abbreviations:

HR, hazard ratio

CI, confidence interval

CDAI, composite dietary antioxidant index

CVD, cardiovascular disease

CKD, chronic kidney disease
